# Quantifying contact lens-related changes in keratoconus corneal topographic indices: an updated Pentacam Scheimpflug imaging analysis

**DOI:** 10.22336/rjo.2022.47

**Published:** 2022

**Authors:** Murat Kasıkcı, Aylin Karalezli, Özgür Eroğul, Hamidu Hamisi Gobeka

**Affiliations:** * Department of Ophthalmology, Mugla Sitki Kocman University Education and Research Hospital, Mugla, Turkey; ** Department of Ophthalmology, Afyonkarahisar Health Sciences University Faculty of Medicine, Afyonkarahisar, Turkey; *** Department of Ophthalmology, Agri Ibrahim Cecen University Faculty of Medicine, Agri, Turkey

**Keywords:** corneal topographic indices, hybrid contact lens, keratoconus, rigid gas-permeable contact lens, Scheimpflug corneal topography, scleral contact lens

## Abstract

**Purpose:** Slowing ectasia progression is critical for maintaining visual potential in keratoconus (KC), for which various therapeutic approaches have been implemented. A Pentacam Scheimpflug imaging device was used to quantify contact lens (CL)-related changes in keratoconus corneal topographic indices.

**Methods:** Thirty KC patients (group 1; 60 eyes) were using one of the three CL (rigid gas-permeable CL (RGPCL)-10, hybrid CL (HCL)-10, and scleral CL (SCL)-10 patients). A control group included 30 KC patients (group 2; 60 eyes) not using CLs due to intolerance or inappropriateness. The Pentacam® HR Scheimpflug imaging device was used to measure topographic indices such as Km anterior, Km posterior, K max, corneal thickness (CT, corneal central, apex, and thinnest), corneal volume (CV), anterior chamber volume (ACV), and anterior chamber depth (ACD) at baseline, 3rd, 6th, and 12th months.

**Results:** The mean ages for groups 1 and 2 were 32±10 and 31±09 years, respectively. Group 1 had a lower but statistically significant change in K max than group 2 (*p*<0.038). Also, group 1 had a minor but non-significant decrease in anterior and posterior keratometry values compared to group 2 (pKm ant. right/ left eye = 0.063/ 0.065 and 0.087/ 0.094, respectively). RGPCL users had significant changes in central CT, thinnest CT and ACD (*p*<0.041). SCL users had more stable changes than other CLs for the thinnest CT along with significant changes in K max, pachy apex and ACV (*p*<0.036). HCL users had significantly higher K max stability (*p*<0.039).

**Conclusion:** Regular use of appropriate therapeutic CLs may help to stabilize corneal deformity, thereby slowing changes in corneal topographic indices in KC.

## Introduction

Keratoconus (KC) is an insidious, usually bilateral but typically asymmetrical progressive corneal ectasia characterized by corneal stroma thinning, epithelial degeneration and Bowman cracks leading to conical corneal shape and protrusion [**1**,**2**]. KC etiology is still largely unexplored [**1**,**3**,**4**], leading to a “two-hit postulation”: a genetic predilection to the corneal disorder and an induction of inflammatory process-related corneal abnormalities. However, various risk factors have been inadequately related, including atopy, several systemic and ocular diseases, and positive family history in 6-8% of patients [**3**,**5**,**6**]. While KC course is extremely unpredictable, conventionally, its onset is likely to be in early adolescence, which may progress later in life.

Corneal topography is a non-invasive technique, commonly used in clinical practice to examine corneal morphology and diagnose KC, allowing many measurement points from the anterior and posterior corneal surfaces [**5**]. Therefore, several topographic evaluation indices enable an accurate and reliable clinical diagnosis.

Treatment of visual impairment secondary to KC, particularly in young adults, has a very high impact on quality of life [**1**,**7**]. The treatment modalities may be either conservative, including spectacle correction and contact lenses (CLs), or surgery. Contact lenses are ocular prosthetic devices used for visual rehabilitation, therapeutic and cosmetic purposes in various corneal disorders and during post-refractive surgery and/ or corneal collagen cross-linking [**8**]. In KC, three different types of CLs, including rigid gas permeable CL (RGPCL), hybrid CL (HCL) and scleral CL (SCL) have been widely used for therapeutic purposes.

The current study was designed to quantify contact lens-related changes in keratoconus corneal topographic indices using a Pentacam Scheimpflug imaging device, as well as to compare data with age- and gender-matched patients who did not use contact lenses.

## Materials and Methods

***Study design and participants***

This observational single-centered study was conducted in the Department of Ophthalmology of Mugla Sitki Kocman University, Faculty of Medicine, between November 2019 and December 2020. The study method complied with the ethical principles laid down in the Helsinki Declaration and was approved by the Institutional Ethics Committee under Decision No. 13/ XII and dated 11/ 11/ 2020. All participants were verbally informed of the study before a written consent was obtained. In case of participants under 18 years old, a written consent was obtained from the parents.

KC patients using RGPCL (10 patients, 20 eyes) (Aeria RGP, LCS, France), HCL (10 patients, 20 eyes) (EyeBrid/ AirKone, LCS, France) or SCL (10 patients, 20 eyes) (ACS Scleral, LCS, France), and a control group (30 patients, 60 eyes) of keratoconus patients not using therapeutic CLs due to intolerance or inappropriateness, were included in the study. However, patients who had visual impairment due to non-KC reasons, had prior ocular surgery and had ocular pathologies, including glaucoma, nystagmus, keratopathy and amblyopia, were not included in the study.

***Ophthalmologic examination and corneal topography***

A routine comprehensive ocular examination was performed in all patients, including measurements of visual acuity in Early Treatment Diabetic Retinopathy Study and Goldmann applanation tonometry intraocular pressure, a slit-lamp biomicroscopy of the anterior and posterior segments, and Scheimpflug corneal topography (Oculus, Germany). In the follow-up examinations, all patients were asked to remove their contact lenses the night before and not to wear them until the measurements were made, to eliminate the short-term effects of CL use on topographic parameters.

Corneal topography consists of a projection and analysis of a luminous reflection that directly illuminates or sweeps the cornea, allowing the examination of its curvature after corneal relaxation. The Pentacam® HR Scheimpflug imaging device was used based on the Scheimpflug principle. Parameters measured during baseline, 3rd, 6th and 12th months included Km anterior, Km posterior, K max, corneal thickness (corneal central, apex and thinnest), corneal volume, anterior chamber volume and anterior chamber depth.

***The Scheimpflug camera working principle***

In normal cameras, the image plane, that is, the sensor, lens plane and object plane in film or digital cameras on the machine are parallel. When a picture of a wall is taken with such a camera, every detail on the wall can be photographed clearly because the image plane is in the focal plane.

**Fig. 1:**
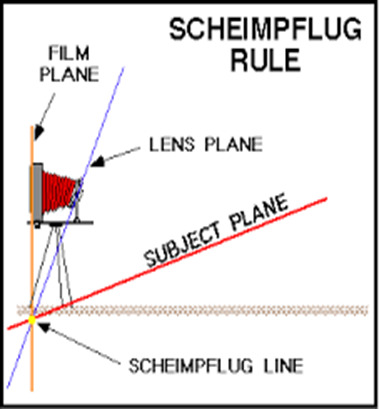
Scheimpflug camera working principle

However, if the object and the image plane are not parallel, the image in the image plane will be clear only in the area where it intersects with the focal plane. In the Scheimpflug cameras, the lens and the image plane are not parallel; instead, the lens is inclined at a certain angle. In this case, flat structures that are not parallel to the image plane can be displayed clearly by taking place entirely within the focal plane. Thus, the Pentacam device takes clear slit images of the cornea, iris and lens using this principle (**Fig. 1**).

***The Pentacam device working principle***

The system is based on the Scheimpflug principle, in which the object plane is not parallel to the image plane. It has a wide focal depth that provides sharp images, containing information from the anterior corneal surface to the posterior lens capsule. It has two cameras, one mounted in the center, which checks fixation and detects pupillary contours and the other mounted on a rotating mechanism, which captures slit images, so that the center of the cornea can be measured precisely.

In addition to the anterior and posterior corneal curvature and height information, pachymetry, corneal aberrations, densitometry and all anterior chamber analyses are provided by the device. The Scheimpflug camera can capture 50 images in less than two seconds. Each image has 500 actual elevation points, so a total of 25000 true elevation points is measured. The Scheimpflug images taken during the examination are digitalized and archived in the computer. After the examination, a three-dimensional model of the anterior segment is created, in which all the anterior segment parameters are also obtained.

The system eliminates artefacts related to eye movements during shooting and provides high resolution imaging of the central cornea. The ability to measure severe corneal irregularities, such as KC, which is too advanced for Placido imaging, makes it possible to calculate pachymetry from limbus to limbus while not affected by tear problems. The Pentacam device differs from the other Topography devices principally because of its ability to evaluate both the front and back surfaces of the cornea.

The Pentacam device combines a rotational Scheimpflug camera and the slit illumination system used in biomicroscopes. Thus, it clearly takes cross-sectional views of the anterior segment of the eye in different axes. While the camera takes images, it rotates in the frontal plane and takes 50 images. It also has a pupil camera that detects eye movements. These images consist of images from the anterior surface of the cornea to the posterior surface of the lens. The pupil must be dilated in almost every case before detection of the lens posterior surface. Then, Scheimpflug distortion is corrected by the Pentacam software, by which the areas in the images are grouped as cornea, iris and lens with image analysis. It is possible to obtain composite anterior segment tomography from these 50 images taken by the camera. The values from this topography are reported with color maps showing, for example, the corneal thickness or dioptric distribution of the cornea. Although the most used reports are corneal pachymetry and dioptric map, it is possible to obtain other information about the anterior segment such as lens opacification and anterior chamber angle with this device. Corneal thickness values and coordinates at three basic points are specified in the report, including the pupil center, the geometric apex of the cornea, and the thinnest point of the cornea. Cornea is accepted as the starting point for the apex cartesian plane. The values on the right side of this point on the horizontal plane are considered positive. The values above this point are taken as positive values in the vertical axis, while the values in the opposite direction are expressed as minus figures.

***Statistical analysis***

Sample size was calculated using the free software G * Power 3.1.9.2 (Franz Foul, University of Kiel, Kiel, Germany). Patients were divided into 4 groups, including the control group. The sample size was calculated as 30 cases (60 eyes) for the control group and 30 cases (60 eyes) for the other groups with 80% power, 0.05 statistical significance level and 0.80 effect size. Descriptive statistics (mean, standard deviation and range), normality test (skewness and kurtosis), one-way analysis of variance (ANOVA) followed by post-hoc Tukey test were performed with Statistica Software v.07. Statistical significance was taken as p <0.05.

## Results

120 eyes of 60 KC patients, 30 of whom (CL users) were using one of the three types of therapeutic CLs in both eyes, were analyzed. The remaining 30 KC patients (non-CL users) were either not suitable or intolerable for CLs. CL users were categorized into three groups: 10 RGPCL users, 10 HCL users, and 10 SCL users. All patients were told to wear their CLs only while lying down. Non-CL users were also assessed on the same day with the other groups.

The mean age of the patients was 32±10 years in CL users and 31±90 years in non-CL users. Male-to-female ratios in CL users and non-CL users were 18:12 and 20:10, respectively. Since the 3rd and 6th month-corneal topographic results were close to the baseline, only changes in the 12th month-results were highlighted in the current study. During the consecutive control periods, the topographic assessment revealed significant positive changes in all indices in both CL users and non-CL users (**Table 1**).

**Table 1:** Changes in topographic indices in keratoconus patients treated with contact lenses versus those who did not use contact lenses

		Contact Lens Users				Non-Contact Lens Users			
Parameters	Side	Baseline	3th Month	6th Month	12th Month	Baseline	3th Month	6th Month	12th Month
Km Anterior	Right	47,08±3,57	47,01±3,55	47,08±3,61	47,15±3,67	46,69±3,77	46,88±3,89	47,07±4,08	47,27±4,33
	Left	47,19±4,37	47,18±4,40	47,12±4,27	47,01±3,94	48,13±3,87	48,25±3,91	48,38±3,95	48,50±3,99
Km Posterior	Right	-7,04±0,89	-7,05±0,90	-7,05±0,94	-7,05±0,96	-6,93±0,84	-6,98±0,85	-7,02±0,86	-7,06±0,88
	Left	-7,05±1,00	-7,05±0,89	-7,04±0,98	-7,02±1,02	-7,22±0,80	-7,24±0,74	-7,27±0,80	-7,29±0,82
K max	Right	52,30±6,07	52,30±6,06	52,28±6,12	52,21±6,17	52,72±6,55	53,05±6,89	53,38±7,23	53,71±7,57
	Left	52,52±7,35	52,48±7,37	52,31±7,22	52,17±6,93	55,60±5,90	55,67±5,96	55,72±6,02	55,75±6,07
Corneal Thickness	Right	466,00±46,80	466,00±46,78	464,00±46,81	462,50±49,89	489,45±36,06	485,64±37,61	481,45±39,16	477,45±40,70
	Left	470,50±42,98	469,54±43,01	468,68±44,01	467,00±44,41	478,10±33,26	476,24±34,03	474,34±34,81	472,41±35,59
Centre Corneal Thickness	Right	454,58±53,05	454,01±53,00	452,01±54,58	450,79±55,77	473,93±36,56	470,27±38,71	466,49±40,85	462,76±42,96
	Left	455,21±51,69	455,00±51,20	453,00±53,27	451,38±54,68	456,62±36,07	453,92±38,02	451,22±39,97	448,52±41,90
Thinnest Local Corneal Thickness	Right	461,83±52,34	460,55±51,78	458±57,69	457,29±55,38	485,00±37,51	480,69±39,17	476,38±41,02	472,07±42,50
	Left	460,92±53,53	460,48±53,65	460,65±53,21	461,08±53,69	469,48±34,45	466,86±35,51	464,24±36,57	461,62±37,63
Apex Corneal Volume	Right	57,03±3,65	57,00±3,55	56,95±3,61	56,88±3,70	57,70±3,34	57,53±3,37	57,35±3,41	57,18±3,44
	Left	57,12±3,39	56,88±3,65	56,85±3,91	56,83±4,32	57,59±3,38	57,47±3,41	57,35±3,44	57,23±3,47
Anterior Chamber Volume	Right	191,83±32,18	192,98±33,20	193,01±32,56	194,00±32,67	182,55±32,65	181,45±33,15	180,42±33,65	179,45±34,15
	Left	189,38±31,26	189,58±31,55	189,96±31,85	190,13±32,16	184,83±35,87	184,11±35,46	183,39±35,05	182,69±34,66
Anterior Chamber Depth	Right	3,21±0,47	3,22±0,35	3,23±0,21	3,24±0,34	3,18±0,31	3,19±0,32	3,19±0,34	3,20±0,33
	Left	3,25±0,38	3,25±0,17	3,24±0,57	3,23±0,39	3,30±0,46	3,30±0,59	3,30±0,51	3,31±0,47

***K max***

**Fig. 2:**
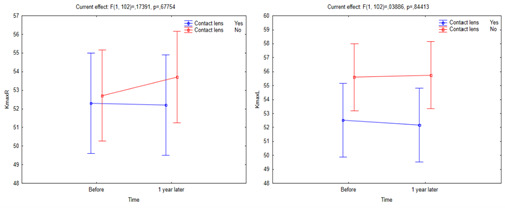
Change in K max value in both eyes of CL users and non-CL users over a one-year follow-up period

 Compared to non-CL users, CL users were associated with a lower but statistically significant change in both eyes in terms of K max (*p*<0.038). However, the upward slope of the red line in the right eye over a short period of 12 months was higher in non-CL users than in CL users (**Fig. 2**).

***Anterior segment parameters***

**Fig. 3:**
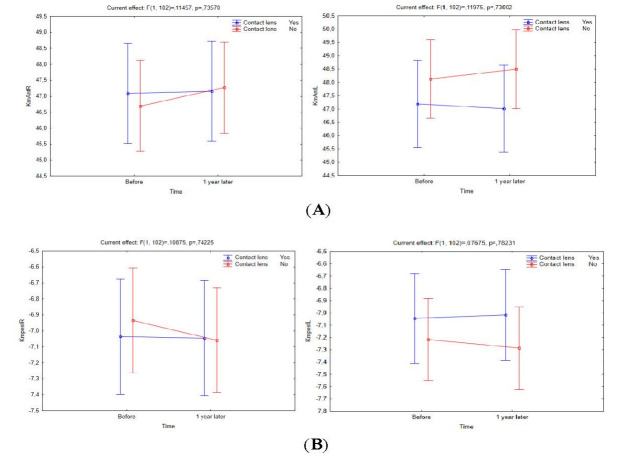
Changes in Km anterior (**A**) and posterior (**B**) parameters in both CL users and non-CL users over a one-year follow-up period

 A one-year follow-up period revealed a slight but statistically non-significant decrease in the anterior and posterior keratometry values in both eyes of CL users relative to non-CL users (*p*Km Ant. right/ left eye = 0.063/ 0.065; and *p*Km Post. right/ left eye = 0.087/ 0.094, respectively). Non-CL users experienced a stiffer acceleration compared to CL users, who experienced a more constant acceleration, implying that the latter group experienced less change in keratometry (**Fig. 3**).

***Anterior chamber depth, anterior chamber volume and corneal volume***

**Fig. 4:**
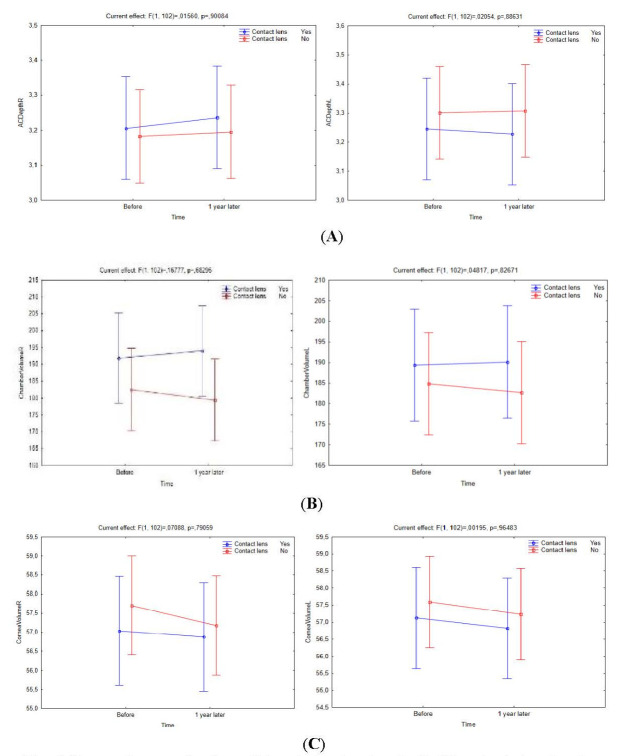
Changes in corneal volume (**A**), anterior chamber depth (**B**) and anterior chamber volume (**C**) between CL users and non-CL users over a one-year follow-up period

 There was no statistically significant difference between CL users and non-CL users regarding the anterior chamber depth (*p*-anterior chamber depth right/ left eye = 0.078/ 0.080), anterior chamber volume (*p*-anterior chamber volume right/ left eye = 0.096/ 0.091), and corneal volume (*p*-corneal volume right/ left eye = 0.094/ 0.086). However, a one-year follow-up of all three groups revealed that non-CL users experienced a greater change in these specific parameters (**Fig. 4**).

***Corneal thickness***

**Fig. 5:**
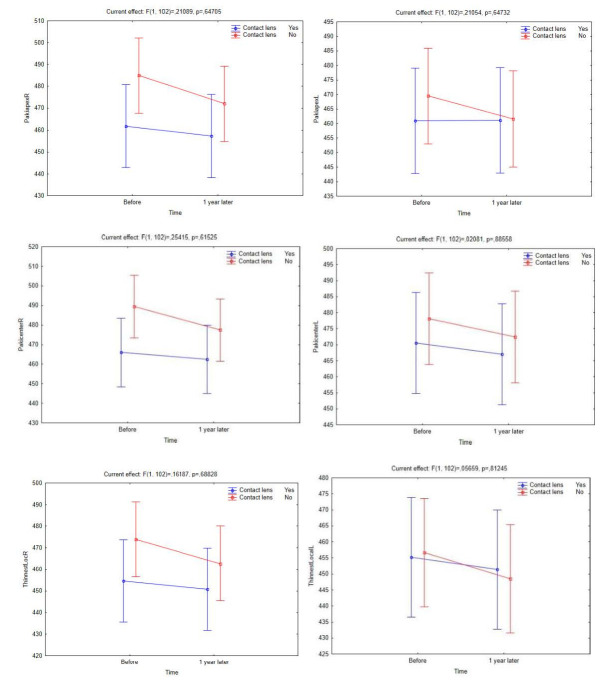
Changes in corneal thickness (apex, centre, thinnest local) in CL users and non-CL users over a one-year follow-up period

 There was no statistically significant difference between CL users and non-CL users in corneal thickness values (*p*Pachy apex right/ left eye = 0.083/ 0.093; *p*Pachy center right/ left eye = 0.074/ 0.085; and *p*Thinnest locale right/ left eye = 0.061/ 0.058). However, **Fig. 5** clearly demonstrates that corneal thickness values changed in favor of more thinning in non-CL users during a one-year follow-up period.

***Intragroup analysis of contact lens users***

**Fig. 6:**
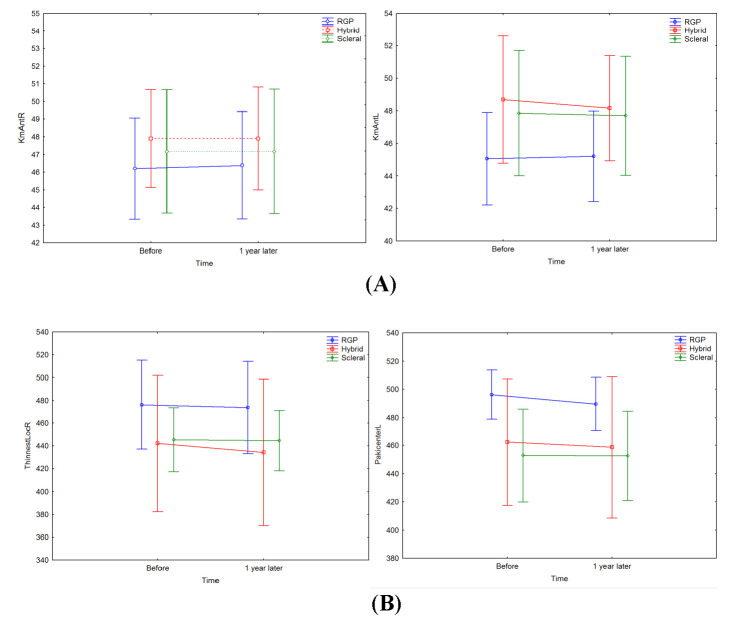
Differences in Km anterior (**A**), thinnest corneal thickness and central corneal thickness (**B**) among patients using different therapeutic contact lenses

 RGPCLs users were associated with statistically significant changes in central corneal thickness (pachy center), thinnest corneal thickness (thinnest locale) and anterior chamber depth parameters during follow-up (*p*<0.041). As a result, more thinning in pachymetry was observed in RGPCL users, which might be ascribed to RGPCL-associated corneal abrasion compared to other types of therapeutic CLs. Change in the thinnest corneal thickness parameter was more stable in SCL users than in other therapeutic CLs. Also, SCL users had statistically significant changes in K max, pachy apex and anterior chamber volume (*p*<0.036). HCL users, however, had statistically significantly more stable K max, pachy apex and thinnest locale parameters (*p*<0.039) (**Fig. 6**).

## Discussion

Clinical use of CLs for visual acuity correction in KC patients was first reported in 1888 [**9**]. Since then, new CL materials and designs have been developed not only to improve visual acuity, the goal being also to improve patient comfort and to reduce changes in corneal topographic indices in relation to this progressive disorder. Various CL types have been widely used to improve visual acuity and reduce changes in corneal topographic indices, which in turn slowed KC progression and thus improved the quality of life in KC patients [**10**]. The current study investigated changes in corneal topographic indices in KC patients treated with RGPCL, HCL, and SCL, and compared them to age- and gender-matched non-CL KC patients. During a one-year follow-up, all patients had topographic changes in favor of KC progression when compared to baseline. Surprisingly, CL users were linked to relatively minor changes in corneal topographic indices, which could be directly linked to the preventive effects of CL regardless of its nature.

In addition to different classification and grading systems, it is important to have a standardized method for documenting corneal ectasia progression. A clinical decision to recommend treatments such as corneal collagen cross-linking focuses solely on treatment that significantly reduces recorded progressive ectasia. According to the Global Consensus on KC and Ectatic Diseases (2015), there is no consistent or clear definition of ectasia progression [**11**]. In addition, it has been stated that KC progression occurs when the anterior and posterior corneal surfaces become steep and the rate of pachymetry change favors thinning. However, it has also been acknowledged that certain quantitative data describing progress were missing [**11**].

Numerous methods for assessing and recording changes in corneal topographic indices in KC patients have been described in literature. Early and newer systems relied solely on serial topographical analysis to record these topographic changes in relation to KC progression [**12**,**13**]. K max anterior (maximum anterior sagittal curvature) is the most commonly used topographic parameter for detecting or recording ectatic progression and is consistently used as an indicator of the collagen cross-linking efficacy [**14**,**15**]. Usually, it represents the steepest anterior corneal curvature taken from a small area [**16**]. This parameter underestimates the degree of ectasia, disregards the contribution of the posterior cornea to progression, and marked ectatic progression may occur without any change or even decrease in K max [**17**]. While one study suggested that K max should be used as a good criterion for the diagnosis of KC progression [**16**], in another study, it was considered a poor parameter for both progression and collagen cross-linking efficacy [**18**].

In the current study, on the other hand, compared to non-CL users, CL users were associated with lower but statistically significant change in both eyes in terms of K max. This was accompanied by a lower rate of KC progression in CL users, which could be linked to a preventive effect of CL on the physiological protrusion of cornea. Given that K max is an important criterion that helps clinicians diagnose KC progression, the current study, which included 120 eyes in a one-year follow-up period, provided valuable statistically significant results for this parameter.

Moreover, the current study revealed a slight but statistically non-significant decrease in the anterior and posterior keratometry values in both eyes of CL users relative to non-CL users. CL users were associated with a more constant acceleration than a stiffer acceleration in non-CL users, suggesting a reduced KC progression in the former group.

The study published by Kanellopoulos et al. [**19**], in which topographic indices derived from the seven anterior surface Pentacam, reported that the index of surface variance and height decentralization could be the most sensitive and specific criteria for the diagnosis and progression of KC. On the other hand, in other studies in which visual acuity, open refraction, and central corneal thickness assessment was performed to track ectatic progression, these parameters were found to be unreliable and did not correlate well with KC severity [**20**,**21**]. Further, several other parameters or systems have been recommended to record progression, including changes in posterior elevation maps and the visual acuity with a decrease in apical corneal thickness or an increase in anterior corneal asymmetry [**19**,**13**]. However, none of these have been validated in literature as methods of assessing progression. They are only limited to the anterior surface of the cornea, or represent a small portion of the cornea, which may not properly indicate changes in the ectatic region. Further, visual acuity methods are highly variable, as many practitioners have determined how unpredictable these subjective measurements can be in KC patients [**19**].

Corneal thickness measurements typically change in favor of thinning after collagen cross-linking, thus limiting its value in recording KC progression [**22**]. It is commonly acknowledged, however, that CLs may induce corneal thickness changes [**23**,**24**] because of corneal structural modification due to decreased basal epithelial cells [**25**]. As far as the current study is concerned, no KC patient needed collagen cross-linking therapy. The investigation was more objectively conducted as to how much the use of therapeutic CLs stabilized corneal thickness in a one-year follow-up period. In this regard, the current study did not reveal any statistically significant difference between CL users and non-CL users with respect to corneal thickness. The reason for the non-significant statistical difference may be attributed to the number of cases and the follow-up period. However, it has been clearly demonstrated that corneal thickness changed in favor of more thinning in non-CL users in a one-year follow-up compared to CL users. Corneal thickness changes in the latter group could be due to CL-related inflammation or an increase in the amount of pro-inflammatory cytokines in tears because of CL use [**26**].

It has been suggested that tomography-derived pachymetry may be a more valuable method for recording ectatic disease and monitoring progression [**27**]. Changes in the posterior corneal curvature [**28**] and corneal asymmetry have also been shown to be additional methods for detecting early KC progression [**19**,**29**,**30**]. Oshika et al. [**31**], on the other hand, assessed global power, regular astigmatism, decentralization, and high-grade irregular astigmatism as a means of measuring the ectasia progression. Moreover, Fourier series harmonic videokeratography and other imaging techniques using Fourier-domain optical coherence tomography have been used to assess KC progression. Optical coherence tomography has been widely used to assess total epithelial thickness, epithelial asymmetry, and biomechanical factors that can be used to record KC progression [**32**]. A wide variety of the proposed KC progression parameters strongly suggests the need for a new or standardized method for recording progression [**11**]. All patients had consistent optical coherence tomography measurements that supported corneal topographic indices. Nonetheless, the current study was unable to find evidence that KC progression had slowed or stopped when optical coherence tomography measurements were compared to changes in the corneal topography indices.

Usually, long-term visual management of KC patients is essential. Factors associated with rapid corneal topographic changes may cause rapid and advanced visual impairment in children with KC. This could potentially have significant negative effects on their social and educational development [**33**]. These patients are treated using a CL correction approach that is like that used in adults in many ways, primarily to improve vision, and provide an appropriately practical, comfortable and physiologically acceptable form of correction. Pradhan et al. [**34**], reported that children can adapt successfully to hard lenses. However, as children often participate in sports activities routinely, lens stability can be an important factor and thus alternative methods are preferred. Rathi et al. [**35**], applied SCLs to children with corneal ectasia and comorbid anterior surface disease to provide effective visual and ocular surface rehabilitation. In the current study, HCLs were applied to the pediatric age group because of the associated improved tolerance. Consequently, this group of patients proved to have a statistically significant more stable K max, pachy apex and thinnest locale parameters compared to other types of CLs.

Contact lenses have been effectively used in KC to decrease corneal irregularity, to form a different regular front surface of the optical system at each disease stage, and consequently to reduce and/ or prevent KC progression [**9**]. For several years, RGPCLs have been the cornerstone of KC correction, particularly at an early disease stage [**36**,**37**]. They generate a thin lacrimal lens between themselves and the cornea, thus correcting the astigmatism caused by the corneal surface irregularities [**9**]. Because traditional RGPCL fittings may be difficult in advanced KC, customized corneal RGPCLs have been used in clinics [**38**]. In the current study, RGPCLs users were associated with statistically significant differences in the central corneal thickness, thinnest corneal thickness, and anterior chamber depth parameters during a one-year follow-up period. When compared to other therapeutic CL types, RGPCL users had more thinning in pachymetry, which could be ascribed to RGPCL-associated corneal abrasion.

Hybrid CLs and SCLs have been recently used for vision correction, as well as decreasing KC progression [**39**]. HCLs are particularly relatively new CL alternatives for KC patients [**40**]. They are preferred to RGPCLs because of their comfort and centralization [**9**]. However, HCLs have a higher incidence of complications that include severe epithelial edema, giant papillary conjunctivitis, corneal vascularization, and circular corneal clouding [**10**,**41**,**42**]. In one study, HCL users were associated with significantly lower keratometry values than the RGPCL users, although visual acuity, corneal thickness and topographic astigmatism values did not differ from the RGPCL users [**43**]. The current study, on the other hand, revealed a statistically significant more stable K max, pachy apex and thinnest locale parameters in HCL users compared to other CLs.

Scleral CLs have benefits for patients with an even more irregular cornea. They increase comfort and visual acuity, and delay the necessity of keratoplasty in advanced KC [**9**]. Scleral CLs may create considerable visual disturbance when the turbidity of the tear meniscus between the cornea and the CL rises. Appropriate ophthalmic solutions should be applied to avoid this complication [**10**]. Overall, SCLs are potent alternatives to RGPCLs and HCLs for visual rehabilitation of complicated corneas [**44**]. Significantly higher keratometry values have been reported in SCL users compared to HCL users [**43**]. Likewise, in the current study, SCL users had more stable keratometry change than other therapeutic CLs in terms of the thinnest corneal thickness parameter. Also, these patients had statistically significant changes in K max, pachy apex and anterior chamber volume, all of which reflect the superiority of these CLs regarding patient comfort, as well as delaying the need for keratoplasty in KC patients.

By providing proper optometric care, especially with CLs to restore functional vision, most KC patients will never need a corneal transplantation. Cassidy et al. [**45**] reported a requirement for corneal grafts in approximately one-tenth of CL users. However, the presence of collagen cross-linking therapy is expected to significantly reduce corneal graft requirements over time. A significant corneal thinning or protrusion that prevents the CL application, CL intolerance, presence of significant corneal scarring and/ or significant risk of corneal perforation are among the ophthalmological referral indications for corneal grafting. The corneal grafting was not required by any CL user in the current study. However, one non-CL user needed corneal grafting due to CL intolerance, which led to a rapid KC progression-related topographic changes and, as a result, to the development of Descemet’s membrane detachment, as well as a severe decrease in visual acuity and other corneal problems.

There are some drawbacks and strengths in this study. Its relatively small study population may be the first and major drawback. Another drawback may be the mean age of the patients, which may reflect a stable stage of topographic changes and KC progression. Further, as the 3rd and 6th month results were close to baseline, only changes in the 12th month results have been emphasized. The comparison of currently three most commonly used CL types in KC patients is one of the strengths of this study. Besides, not only has an assessment been carried out between CL users and non-CL users, intragroup analysis of therapeutic CL variants has also been done. As a result, the authors are adamant that the current study is the first of its kind to investigate KC patients in terms of changes in corneal topographic indices and, indirectly, KC progression between CL users and non-CL users, as well as among CL variants. Nonetheless, even more prospective studies with relatively large cohorts are required to investigate how and to what extent different CL types may subsequently affect corneal topographic indices in KC patients. Although it was emphasized in our study that the use of contact lenses could stop the progression of keratoconus, it should be supported by studies with a longer follow-up period. Moreover, our study lacked comparisons with patients treated with CXL. The evaluation of the data resulted by comparing the data obtained with the use of contact lenses and the patient population receiving CXL treatment will guide in terms of investigating the effect of contact lenses in the treatment of keratoconus.

## Conclusion

In conclusion, the use of therapeutic CLs in KC patients improved the stability of corneal topographic indices. RGPCL users particularly had more thinning in pachymetry. Scleral CL usage was linked to more stable corneal topographic changes. Significant stability in K max, pachy apex, and thinnest corneal thickness were found in hybrid CLs. These findings imply that stabilizing corneal deformity and reducing changes in corneal topographic indices by using appropriate therapeutic CLs on a regular basis may ultimately result in more than just a slowing of the rate of KC progression. This could also help KC patients with visual rehabilitation by avoiding or delaying the need for corneal grafting.

**Conflict of Interest statement**

The authors claim no conflict of interest. Both authors certify that they have no association or participation with any organization or individual with any financial interest or non-financial interest in the subject matter or materials discussed in this article.

**Informed Consent and Human and Animal Rights statement**

All participants were verbally informed of the study before a written consent was obtained. In case of participants under 18 years old, a written consent was obtained from the parents. 

**Authorization for the use of human subjects**

Ethical approval: The study method complied with the ethical principles laid down in the Helsinki Declaration and was approved by the Institutional Ethics Committee under Decision No. 13/ XII and dated 11/ 11/ 2020. 

**Acknowledgements**

None.

**Sources of Funding**

This research did not receive any specific grant from funding agencies in the public, commercial, or non-profit sectors.

**Disclosures**

None.
